# Sleep Basic Research on Verifying the Effects of Natural Compounds on Wakefulness or Sleep

**DOI:** 10.14789/jmj.JMJ21-0022-R

**Published:** 2022-02-16

**Authors:** SHOHEI NISHIMON

**Affiliations:** 1 Department of Psychiatry and Behavioral Science, Juntendo University Graduate School of Medicine, Tokyo, Japan; 1 Department of Psychiatry and Behavioral Science, Juntendo University Graduate School of Medicine, Tokyo, Japan

**Keywords:** sleep, basic research, mouse, ginkgolide, sake yeast

## Abstract

I studied at the Sleep and Circadian Neurobiology Laboratory, Department of Psychiatry and Behavioral Sciences, Stanford University School of Medicine, from April 2018 to March 2020. At Stanford University, I mainly researched the following themes: (1) sleep basic research using mice (administering compounds with sleep or wakefulness effects to mice and examining their effects), and (2) research on circadian rhythm disorders. There are only a few institutions in the world that can conduct sleep basic research using mice, and Stanford University is a wonderful environment to immerse yourself in research, as it is home to not only psychiatrists but also neurologists and many basic researchers. In this article, I would like to review the experiments I conducted during my study abroad, using mice to verify the effects of natural compounds on wakefulness or sleep. In one study, we evaluated the effects of ginkgolides (A, B, and C) and bilobalide on arousal, locomotion, and core body temperature. The results showed that only ginkgolide B dose-dependently increased the amount of arousal and decreased the amount of NREM sleep in the physiological sleep-wake cycle of mice. In another study, we tested the sleep-inducing effects of sake yeast in mice under an acute insomnia model. We showed that sake yeast dose-dependently increased REM and non-REM sleep, decreased arousal within 6 hours after oral administration of sake yeast, and decreased locomotion and core body temperature in a new cage.

## Introduction

I studied at the Sleep and Circadian Neurobiology Laboratory, Department of Psychiatry and Behavioral Sciences, Stanford University School of Medicine, USA from April 2018 to March 2020. At Stanford University, I mainly researched the following themes: (1) sleep basic research using mice (administering compounds with sleep or wakefulness effects to mice and examining their effects), and (2) research on circadian rhythm disorders. With the support of many people, I have been able to publish four papers as the first author^1-4)^. In this article, I would like to review two experiments I conducted during my study abroad, in which I investigated the effects of natural compounds on wakefulness or sleep in mice. One is a validation experiment on the arousal effects of ginkgolides (A, B, and C) and bilobalide, which are terpene lactones contained in *Ginkgo biloba* extract. The other is an experiment to verify whether sake yeast, which has a sleep-inducing effect, can similarly induce sleep under an acute insomnia model. First, I would like to explain the surgical procedure for measuring sleep electroencephalography (EEG), electromyography (EMG), locomotion, and core body temperature of mice used in the experiment^2, 4)^.

## Experimental Procedure for Measuring Sleep in Mice

### Surgical procedures for telemetry implant and headstage

A telemetry implanting device (G2 E-Mitter; Mini Mitter OR, Oakmont, PA, USA) was intraperitoneally implanted under 3% isoflurane anesthesia to evaluate the core body temperature and locomotor activity. The mice were surgically prepared for EEG and EMG recordings with a headstage attached to the cable recorder. Two of the four electrodes, which consisted of stainless-steel screws, were implanted into the skull 1.5 mm lateral and 1.5 mm anterior to the bregma (over the motor cortex). The other two electrodes were implanted 3 mm lateral and 1 mm anterior to the lambda (over the visual cortex) for the EEG. Two Teflon-coated stainless-steel wires were inserted into the neck extensor muscles on both sides for the EMG. The six electrodes were attached to one 2 × 3 pin header that was secured to the skull with dental acrylic. Postoperatively, the mice subcutaneously received an analgesic (carprofen, 3 mg/kg) and antibiotic (enrofloxacin, 25 mg/kg), and were allowed to recover for 2 weeks before the experiments.

### The Procedures of data collection

After 2 weeks of the postoperative recovery period, the mice were individually moved into experimental cages specifically modified to include plastic micro- isolation cages fitted with a low-torque slip-ring commutator (Biella Engineering, Irvine, CA, USA). Each cage was placed in the custom-designed recording chamber with individual ventilated compartments. The following day, the headstages of the mice were connected to the EEG/EMG recording cables, which consisted of the slip ring commutator through a 15-20 cm of lightweight six-strand shielded signal cable (NMUF6/30-4046SJ; Cooner Wire, Chatsworth, CA, USA). The commutator output was amplified. The mice had ad libitum access to food and water in the experimental cages. The room temperature was maintained at 23°C ± 1°C. A 12-h light/dark cycle was implemented throughout the experiment. After 1 week of habituation to the experimental conditions, consecutive EEG/EMG, core body temperature, and locomotor activity recordings were performed.

The EEG/EMG signals were amplified using a Grass Instrument model 12 (West Warwick, RI, USA), and digitally filtered (30 Hz Low Pass Filter for EEG; 10-100 Hz Band Pass Filter for EMG). Subsequently, the EEG signals were captured at 256 Hz using data acquisition software (Vital Recorder; Kissei Comtec Co. Ltd., Matsumoto, Japan). EEG signals collected with ipsilateral bipolar EEG electrodes placed over motor and visual cortices, together with the bipolar EMG signals, were recorded for sleep scoring.

### Sleep recording

**Figure 1:**
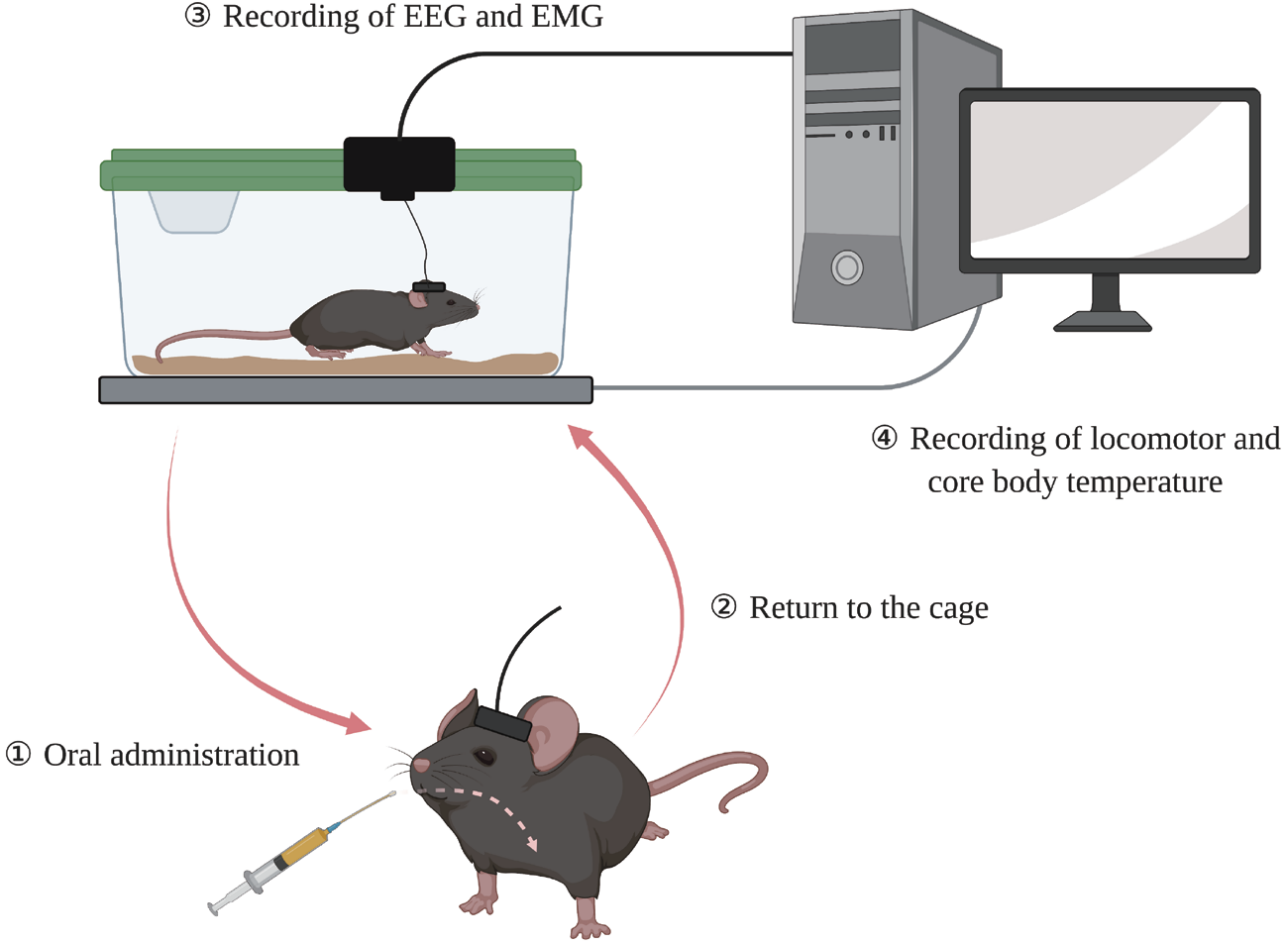
After the headstage installation and abdominal transmitter implantation, the mice are observed in the cage for several days. After that, the experiment will be initiated. First, the mice are taken out of the cage and orally administered the compound. After administration, the mice are immediately returned to their habituated cages. Then, EEG and EMG measurements, locomotor activity and core body temperature measurements will be conducted.

We visually confirmed the sleep/wake stages based on EEG and EMG signals in 10-s epochs, according to our standard criteria using the Sleepsign software (Kissei Comtec Co. Ltd.) (Figure 1). We required 50% or more of a specific state in each epoch to score the epoch. Firstly, wakefulness was characterized by desynchronized, low-amplitude, and mixed-frequency (>4 Hz) EEG with high EMG activity, which appears as a rhythmic theta/alpha (7-9 Hz) wave. Secondly, non-rapid eye movement (NREM) sleep was characterized by synchronized, high-amplitude, and low-frequency (0.25-4 Hz) EEG with low EMG activity. Thirdly, similar to wakefulness, rapid eye movement (REM) sleep was characterized by desynchronized, low- amplitude, and mixed-frequency EEG, whereas the EMG activity during REM sleep was low compared with that observed during NREM sleep. During REM sleep, some muscle fasciculation may be observed in the EMG trace. Typically, rhythmic theta/alpha (7-9 Hz) waves with reduced EMG activity are dominant. Changes in the sleep state were considered when at least one 10-s epoch was scored as appearing a different sleep stage, and the duration of the state episode was determined as the duration of a continuous single state episode. All sleep scoring was performed by a single observer who was blinded to animal information.

### Core body temperature and locomotor data analyses

The plastic cages were individually placed on the telemetry receiver (Series 4000, Mini Mitter) that transmitted the signals for core body temperature and locomotor activity every 1 min (Figure 1). Subsequently, the data were acquired using the Vital View software (Mini Mitter, OR). The 0.5- hourly mean value of core body temperature (°C) after the injection of compounds and vehicle was first calculated. Then, the difference of temperature between before (baseline) and after the injection was assessed and plotted with a 0.5-h interval for 6 h to adjust the variability of baseline. Furthermore, the area under the curve was also assessed in each compound group for 6 h after injection. The mean value of locomotor activity (counts/h) for 6 h after the injection was assessed and plotted with a 1-h interval. The cumulative amount (counts/h) for 6 h after injection was also assessed and plotted with a 1-h interval.

## Ginkgolide B, the major active compound in *Ginkgo biloba*, dose-dependently increases wakefulness and decreases NREM sleep

Generally known as Ginkgo, *Ginkgo biloba* is widely used in traditional medicine and for food. EGb 761, extracted from *Ginkgo biloba* leaves, is known as a therapeutic agent and dietary supplement for cerebrovascular and neurodegenerative diseases due to its antioxidant, anti-inflammatory, and neuroprotective properties, while EGb 761 has also been shown to relieve anxiety symptoms in patients with generalized anxiety disorder and adjustment disorder without sedation. EGb 761 is composed of flavonoid glycosides and terpene lactones. Of the terpene lactones, ginkgolides account for 2.8% to 3.4% and virobalides 2.6% to 3.2%. Ginkgolides exert neuroprotective effects against ischemic stroke caused by neuronal damage, and inflammatory and neurodegenerative diseases such as dementia and Alzheimer's disease. In particular, ginkgolide B is the most potent selective and competitive platelet-activating factor receptor antagonist, and has antioxidant and anti-inflammatory effects.

In addition, ginkgolides (A, B, C) and bilobalide have been shown to antagonize gamma-aminobutyric acid type A (GABAA) receptors and glycine receptors in the central nervous system. We assessed the effects of ginkgolides (A, B, C) and bilobalide on vigilance, locomotor, and core body temperature using EEG, EMG, and telemetry implants. We intraperitoneally injected two doses of ginkgolides A, B, C or bilobalide (i.e., 0.5 mg/kg and 5.0 mg/kg) and vehicle on different days *Zeitgeber Time* (ZT)2.

As a result, we showed that only ginkgolide B dose-dependently increased the amount of wakefulness and decreases the amount of NREM sleep during the physiological sleep-wake cycle in mice. These effects were thought to be obtained by negatively modulating the activities of GABA and glycine at GABAA and glycine receptors, respectively^2)^.

## Effects of sake yeast on sleep promotion in mice with stress-induced acute insomnia

Conventional benzodiazepines, non-benzodiazepine sedative-hypnotics, melatonin receptor agonists, and orexin receptor antagonists are used in general clinical practice for drug treatment of insomnia. However, there are well-known adverse risks associated with the use of benzodiazepines and non-benzodiazepines sedatives, including tolerance, addiction, abuse, and paradoxical reactions such as aggression, violence, and impulsivity. In addition, the use of benzodiazepines and non-benzodiazepines has been associated with an increased risk of falls, fractures, and cognitive impairment in the elderly.

Recent studies have shown that several natural compounds that focus on adenosine receptor (AR) activation can have sleep-promoting effects. Previous studies have shown that sake yeast is a dose-dependent adenosine A_2A_ receptor (A_2A_R) agonist that promotes NREM sleep by accumulating S-adenosylmethionine or methylthioadenosine during wakefulness in mice. Similarly, some previous studies have demonstrated that sake yeast can improve sleep quality in humans. Based on these previous studies, in the present study we tested the sleep-inducing effects of sake yeast in mice under an acute insomnia model.

In this study, we evaluated the effects of sake yeast, an adenosine analogue, on sleep-wake stage, locomotion, and core body temperature in a stressful environment as a model of acute insomnia using sleep EEG, electromyography, and telemetry implants in mice. We have already verified in previous experiments that a novel environment in which mice in the sleep phase are transferred from habituated cages to new cages is useful as a model for acute insomnia^5)^. In the present study, two different doses of sake yeast (200 mg/kg and 300 mg/kg) and vehicle without sake yeast at ZT2 on different days were orally administered to mice removed from habituated cages and then transferred to new cages.

As a result, we showed that sake yeast dose-dependently increased REM and non-REM sleep, decreased arousal within 6 hours after oral administration of sake yeast, and decreased locomotion and core body temperature in a new cage. These results indicate that sake yeast may induce sleep even under conditions of acute insomnia via activation of A_2A_R. In the future, it will be interesting to investigate the potential involvement of adenosine in the pathophysiology of insomnia^4)^.

## Conclusion

We found that ginkgolide B dose-dependently increased the amount of arousal and decreased the amount of NREM sleep in mice. We also showed that sake yeast regulates locomotion and thermoregulation and promotes both REM and NREM sleep in acute insomnia via activation of A_2A_R. Understanding the mechanisms by which these natural compounds induce wakefulness or sleep effects may provide an opportunity to develop new therapeutic agents. However, in both studies, there have been no comparative studies with existing synthetic drugs that have wakefulness- or sleep-inducing effects, and further validation is desirable.

## Funding

No funding was received.

## Author contributions

SN drafted the report, and contributed to and have approved the final manuscript.

## Conflicts of interest statement

The author declares that there are no conflicts of interest.
